# Reduction in *mdx* mouse muscle degeneration by low-intensity endurance exercise: a proteomic analysis in quadriceps muscle of exercised compared with sedentary *mdx* mice

**DOI:** 10.1042/BSR20150013

**Published:** 2015-06-22

**Authors:** Simona Fontana, Odessa Schillaci, Monica Frinchi, Marco Giallombardo, Giuseppe Morici, Valentina Di Liberto, Riccardo Alessandro, Giacomo De Leo, Vincenzo Perciavalle, Natale Belluardo, Giuseppa Mudò

**Affiliations:** * Department of Biopathology and Medical Biotechnology, Section of Biology and Genetics, University of Palermo, I-90127 Palermo, Italy; † Department of Experimental Biomedicine and Clinical Neurosciences, Section of Physiology, University of Palermo, I-90134 Palermo, Italy; ‡ Department of Biomedical and Biotechnological Sciences, Section of Physiology, University of Catania, I-95125 Catania, Italy

**Keywords:** carbonic anhydrase, exercise, muscle oxidative stress, muscle proteomic, muscular dystrophy, X-linked muscular dystrophy (*mdx*)

## Abstract

In our recent study was shown a significant recovery of damaged skeletal muscle of mice with X-linked muscular dystrophy (*mdx*) following low-intensity endurance exercise, probably by reducing the degeneration of dystrophic muscle. Consequently, in the present work, we aimed to identify proteins involved in the observed reduction in degenerating fibres. To this end, we used proteomic analysis to evaluate changes in the protein profile of quadriceps dystrophic muscles of exercised compared with sedentary *mdx* mice. Four protein spots were found to be significantly changed and were identified as three isoforms of carbonic anhydrase 3 (CA3) and superoxide dismutase [Cu-Zn] (SODC). Protein levels of CA3 isoforms were significantly up-regulated in quadriceps of sedentary *mdx* mice and were completely restored to wild–type (WT) mice values, both sedentary and exercised, in quadriceps of exercised *mdx* mice. Protein levels of SODC were down-regulated in quadriceps of sedentary *mdx* mice and were significantly restored to WT mice values, both sedentary and exercised, in quadriceps of exercised *mdx* mice. Western blot data were in agreement with those obtained using proteomic analysis and revealed the presence of one more CA3 isoform that was significantly changed. Based on data found in the present study, it seems that low-intensity endurance exercise may in part contribute to reduce cell degeneration process in *mdx* muscles, by counteracting oxidative stress.

## Abstract (precis)

By proteomic analysis we found an up-regulation of four carbonic anhydrase-3 (CA3) isoforms and a down-regulation of superoxide dismutase [Cu-Zn] (SODC) in quadriceps of sedentary X-linked muscular dystrophy (mdx) mice as compared with wild–type (WT) mice and the levels were significantly restored to WT values following low-intensity endurance exercise.

## INTRODUCTION

Duchenne muscular dystrophy (DMD) is a lethal X-linked muscle disease due to a defect in the sub-sarcolemmal protein dystrophin, which leads to membrane fragility, muscle necrosis, motor weakness, myofibre death and replacement of skeletal muscle by fibrous and fatty connective tissue, due to failed regeneration [1]. In patients with DMD, muscle biopsy characteristically demonstrates areas of necrotic or degenerating muscle fibres, often observed in clusters surrounded by macrophages and lymphocytes and areas with small, immature, centrally-nucleated fibres reflecting muscle regeneration from myoblasts [2]. Over the last few years, MS-based proteomics has been successfully applied to investigate normal and pathologically-altered skeletal muscle tissue [3], including the proteomic profiling of muscle tissues from *mdx* mice that has revealed differential degrees of perturbed protein expression patterns in dystrophin-deficient fibres [4]. Many of these proteomic analyses on muscles of mice with X-linked muscular dystrophy (*mdx)* and DMD have been performed with the aim of unravelling the molecular pathogenesis of muscular dystrophy [5] and these analyses discovered several proteins that were differentially expressed in dystrophic muscles [6]. This large number of proteins change has hampered the possibility of developing therapeutic approach to muscular dystrophy by targeting proteins modified.

The lack of dystrophin in *mdx* mice leads to cycles of muscle degeneration and regeneration processes and various strategies have been proposed in order to reduce the muscle-wasting component of muscular dystrophy [7,8], including implementation of an exercise programme. In our recent study, it was shown that following low-intensity endurance exercise, there was a significant recovery of damaged skeletal muscle in *mdx* mice, probably by reducing the degeneration of dystrophic muscle [9]. Consequently, in the present work, we aimed to identify proteins involved in the observed reduction in degenerating fibres by using proteomic analysis to evaluate changes in the protein profile of quadriceps dystrophic muscles of exercised *mdx* compared with sedentary *mdx* mice.

## MATERIAL AND METHODS

### Preparation of skeletal muscle proteins

The present study was performed using the same quadriceps muscle samples of experimental mice groups used in our previous work [9] in order to better compare results of proteomic analysis with previous data showing a reduction in degeneration process in mice subjected to low-intensity endurance exercise. Therefore, muscle samples used in the present study are referred to experimental condition of our previous work [9] in which the following mice and experimental groups were used: male *mdx* mice (C57BL/10ScSn-Dmd*^mdx^*/J from Jackson Laboratories) 8-weeks-old, divided in sedentary *mdx* (MDX-Sed) mice or exercised *mdx* (MDX-Ex) mice and C57/BL wild-type (WT) mice, divided in sedentary WT (WT-Sed) and exercised WT (WT-Ex), used as control mice. The training protocol, using motorized rotating treadmill (Rota-Rod; Ugo Basile, Biological Research Apparatus) for low-intensity endurance exercise, is described in Frinchi et al. [9]. Shortly, mice ran 5 days/week for 6 weeks: the first 2 weeks all exercised mice underwent a period of acclimatization with very low speed (16 rotations/min) for 15 min during the first week and for 30 min during the second week [9]. This acclimatization trial was followed by 30 days of low-intensity endurance training with increasing duration (session time) and intensity (rotation time) of weekly training (Table 1). For the present study, we used quadriceps muscles derived from mice groups killed following 30 days of training. Thick cryosections (60 μm), sampled at five different levels, were prepared from muscles of three mice for each group and used for protein extraction [10]. The sampled muscle sections were homogenized in cold buffer containing 50 mM Tris/HCl, pH 7.4, 150 mM NaCl, 1% triton, 0.5% SDS, H_2_O and protease inhibitor cocktail Sigma–Aldrich S.r.l.). The homogenate was left on ice for 30 min and then centrifuged at 25 000 ***g*** for 30 min at 4°C. The supernatants were stored at −80°C and aliquots were taken for protein determination. All procedures involving animals and their care were conducted in conformity with the Italian institutional guidelines (D.L. no. 116, G.U., suppl. 40, February 18, 1992 and D. LGS. no. 26, GU n.61, March, 2014).

**Table 1:** Over/underexpressed proteins in WT and *mdx* quadriceps NDE, no differential expression.

Nr Spot	Protein name	AC number*	pI/*M*_r_ (kDa)	MDX-Sed compared with WT-Sed^†^		MDX-Ex compared with MDX Sed^†^	
				Fold change	*P-*values	Fold change	*P-*values
1	MYH4	Q5SX39	5.37/59.1	−4.3	0.027	NDE	–
2	TnT	Q9QZ47	8.65/33.7	−3.1	0.020	NDE	–
3	CA3	P16015	6.72/28.1	2.8	0.046	−3.6	0.022
4	CA3	P16015	6.74/27.6	2.7	0.040	−2.3	0.042
5	CA3	P16015	6.82/27.8	2.1	0.033	−1.7	0.039
6	MLRS	P97457	4.78/15.8	−7.9	0.005	NDE	–
7	SODC	P08228	6.10/14.6	−5.28	0.02	2.10	0.01
8	α/PVALB	P32848	4.84/12.9	−4.7	0.013	NDE	–

*Accession number in UniProtKB/Swiss-Prot

†Only proteins spots exhibiting at least 1.5-fold changes in %Vol and *P*<0.05 (calculated by Student's *t* test) were considered differentially expressed. Differences with *P*<0.05 were considered significant.

### 2D gel electrophoresis and image acquisition and analysis

2D gel electrophoresis (2DE) was carried out in the IPGphor system (GE Healthcare) as described by Fontana et al. [11]. Analytical gels were stained with ammonium silver nitrate [12] and preparative gels with Coomassie Blue.

Stained gels were scanned by a ImageScanner II (GE Healthcare) previously calibrated with a greyscale marker (Kodak), digitalized with Labscan 5.00 (v 1.0.8) software (GE Healthcare) and analysed with the ImageMaster™ 2D Platinum v 6.0 software (GE Healthcare). Within each group (WT-Sed, MDX-Sed and MDX-Ex) three silver stained gels obtained from three independent experiments were analysed to guarantee representative results. After automated spot detection, spots were checked manually to eliminate any possible artefacts, such as streaks or background noise. Biological replicates showed less than 10% of variability in the number of protein spots detected. The patterns of each sample were overlapped and matched, using landmark features, to detect potential differentially expressed proteins. The gel presenting the highest spot number was selected as master gel and the coefficient of determination (r2) for each gel pair was calculated in order to ensure the analyses reliability (pair average r2: 0.85). In order to reduce experimental error, quantitative analysis was based on evaluation of relative spot volume (%V=V single spot/V total spots, where V is the integration of the optical density over the spot area). In the comparison of MDX-Sed mice compared with WT-Sed mice and of MDX-Ex mice compared with MDX-Sed mice, proteins were considered differentially expressed when the ratio of the %Vol average (three gels) was higher than 1.5 and the *P*-value deduced from the *t* test was <0.05.

### In-gel digestion and protein identification by MALDI–TOF/TOF–MS

Coomassie Blue-stained protein spots were excised from the preparative gels and cut into 1-mm pieces. In-gel digestion was performed as described by Shevchenko et al. [13] with minor modifications [11].

The digested peptides were desalted and cleaned with ZipTip C18 pipette tips (Millipore Corp.) before obtaining the mass spectrum of the peptide mixture. All analyses were performed using a Bruker Daltonics Autoflex (Bruker Daltonics) operated in the delayed extraction and reflectron mode with the following parameters: 20 kV acceleration voltage, 95% grid voltage, 100 ns delay time and 500 *m/z* low-mass gate. For acquisition of a mass spectrometric peptide map, a 1-μl aliquot from the peptide extracts was premixed with 1 μl of matrix (10 mg/ml α-Cyano-4-hydroxycinnamic acid (CHCA) in 35% acetonitrile (ACN) and 0.1% trifluoroacetic acid (TFA)) and spotted on to a MALDI target plate. Measurements were externally calibrated with a standard peptide mixture (Peptide Calibration Standard, Bruker Daltonics). Both MS and MS/MS data were acquired with a N_2_ laser at a 25-Hz sampling rate. The monoisotopic masses were processed for identification. For MS/MS spectra, the peaks were calibrated by default and smoothed. Data were analysed using MASCOT software (Matrix Science), by searching against the Swiss-Prot/UniprotKB database. Taxonomy was limited to *Mus musculus* and the following search parameters were used: enzyme, trypsin; allowed missed cleavages, carbamidomethyl cysteine as fixed modification by the treatment with iodoacetamide; variable modifications, oxidation of methionine; mass tolerance for precursors was set to 50 ppm and for MS/MS fragment ions to ±0.5 Da. The identifications were accepted if the extent of sequence coverage was at least 10% and the number of matched peptides was at least 5. Only individual scores greater than 55, defined by MASCOT probability analysis (*P* ≤ 0.05) were accepted.

### Validation of proteomic data by western blotting analysis

For 1D-western blotting (WB), 20 μg of protein extracts were separated by SDS/PAGE (12% gel) and transferred on to Hybond ECL Nitrocellulose Membrane (GE Healthcare Life Sciences). The membrane was incubated in blocking solution (5% non-fat dry milk, 20 mM Tris, 140 mM NaCl, 0.1% Tween-20) and probed overnight at room temperature with specific antibodies against superoxide dismutase [Cu-Zn] (SODC; Cell Signaling Technology) and β-actin [14]. After incubation with blocking solution, the membrane was probed overnight at room temperature with specific antibodies against carbonic anhydrase-3 (CA3; Cell Signaling Technology).

The results were confirmed by at least three independent experiments. The blots were subjected to densitometric analysis by using Image J (1D-WB) or ImageMaster 2D Paltinum 6.0 (2D-WB) software. For the quantification, the intensity of the immunostained bands/spots corresponding to SODC and to CA3 were normalized with the bands/spots corresponding to the β-actin from the same gel. Statistical analysis of the data was performed by Student's *t* test; *P*-values ≤ 0.05 were considered statistically significant.

## RESULTS

### Proteome profile characterization of muscle quadriceps

Since previous data [9] showed that both in gastrocnemius and in quadriceps muscle there was remarkably significant reduction in inflammatory-necrotic areas in *mdx* mice exercised for 30 days, in the present work we decided to restrict the proteomic survey to the experimental mice groups exercised for 30 days and to focus our attention to quadriceps muscle. Quadriceps proteins purified from WT-Sed, WT-Ex, MDX-Sed and MDX-Ex mice were analysed by 2DE analysis. Although 2D gels of muscle fibre homogenates do not represent the entire muscle protein complement, optimized electrophoretic procedures can separate a large and representative proportion of key metabolic, structural, regulatory and contractile elements [15]. Therefore, this procedure is the most powerful tools for conducting comparative proteins profiling investigations, although very low-density components and proteins with extreme pI values may be under-represented in 2D gels [16].

As shown in the Supplementary Figure S1, the silver stained proteomic maps of each group of mice revealed an overlapping spot pattern within the range of pI 4–8 and *M*_r_ from 150 to 9 kDa. In order to validate the quality of our extracts, we compared the spot pattern of our samples to the reference proteomic maps of muscle tissues. We found that the proteome profile of our extracts was comparable to well-characterized 2DE maps of mouse and rat gastrocnemius, soleus, vastus lateralis and diaphragm (see: http:://world-2dpage.expasy.org/swiss-2dpage/viewer) [17]. To further characterize the protein profile of quadriceps, we performed a MALDI-TOF/TOF-MS analysis and using the Mascot search program we identified 36 protein spots randomly selected in the sedentary WT-Sed mice 2DE gel. Moreover, by WB, we identified eight isoforms of β-actin (Supplementary Figure S2). Altogether, we identified 44 protein spots corresponding to 17 different proteins, marked with their UniProt accession number in Figure 1 and listed by this number in the Supplementary Table S1, where the protein name, pI-values and relative molecular masses, identification method, the number of matched peptide sequences, percentage sequence coverage and Mascot score are also reported. All identified proteins in the quadriceps of WT and MDX mice confirm previous available data from proteomics map of several types of muscle tissue [4,17–19].

**Figure 1:**
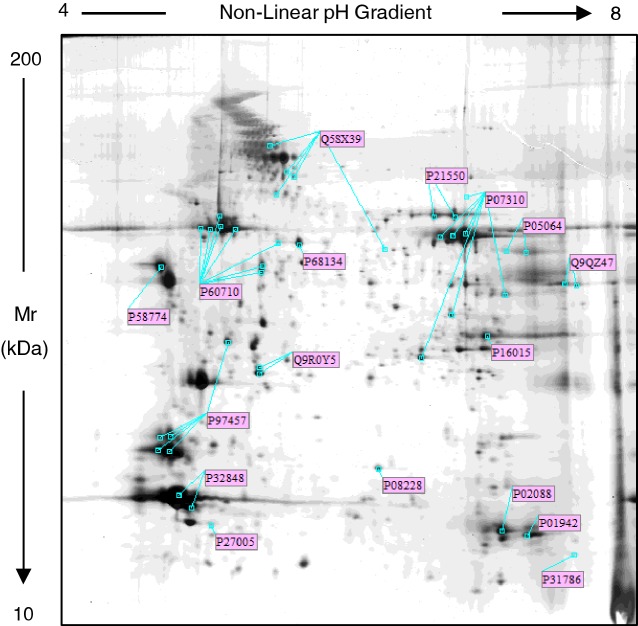
Representative silver stained proteomic maps of quadriceps from sedentary WT mouse Proteins (60 μg) were loaded on IPG strips (18 cm, 3.5–10 non-linear pH gradient). The second dimension was performed on a vertical linear-gradient slab gel (9%–16%). The pH-values of the first dimension gel system and molecular mass range (in kDa) of the second dimension are indicated on the top and on the left of the panels respectively. Spots labelled with the UniProt Accession Number were identified by MALDI-TOF/TOF-MS. Details of the identification are reported in Supplementary Table 1, supporting information.

### Comparative proteomic analysis of 2DE maps of quadriceps from WT and *mdx* mice

To investigate the potential proteomic alterations induced by low intensity endurance exercise in *mdx* mouse muscles, proteome profile of quadriceps isolated from WT-Sed and MDX-Sed or MDX-Ex mice were compared by using Image Master 2D Platinum 6.0. As first step, we searched variation in protein expression profile of quadriceps in MDX-Sed mice as compared with WT-Sed mice. We found that eight protein spots showed significant differences (*P*<0.05) in %Vol (Figure 2). Among these eight proteins, seven were identified by MS and the protein corresponding to spot 4, for which the MS failed the identification, was identified by WB. Based on its position in the map, we hypothesized that the spot 4 could represent an isoform of CA3. As shown in Supplementary Figure S3, WB analysis confirmed the identity of this spot. The identities and fold-changes of all eight modulated protein spots are listed in Table 1. These data showed that the proteins altered in quadriceps of MDX-Sed belonged to six classes of cellular components involved in muscle contraction and in cellular oxidative stress response (CA3 and SODC).

**Figure 2:**
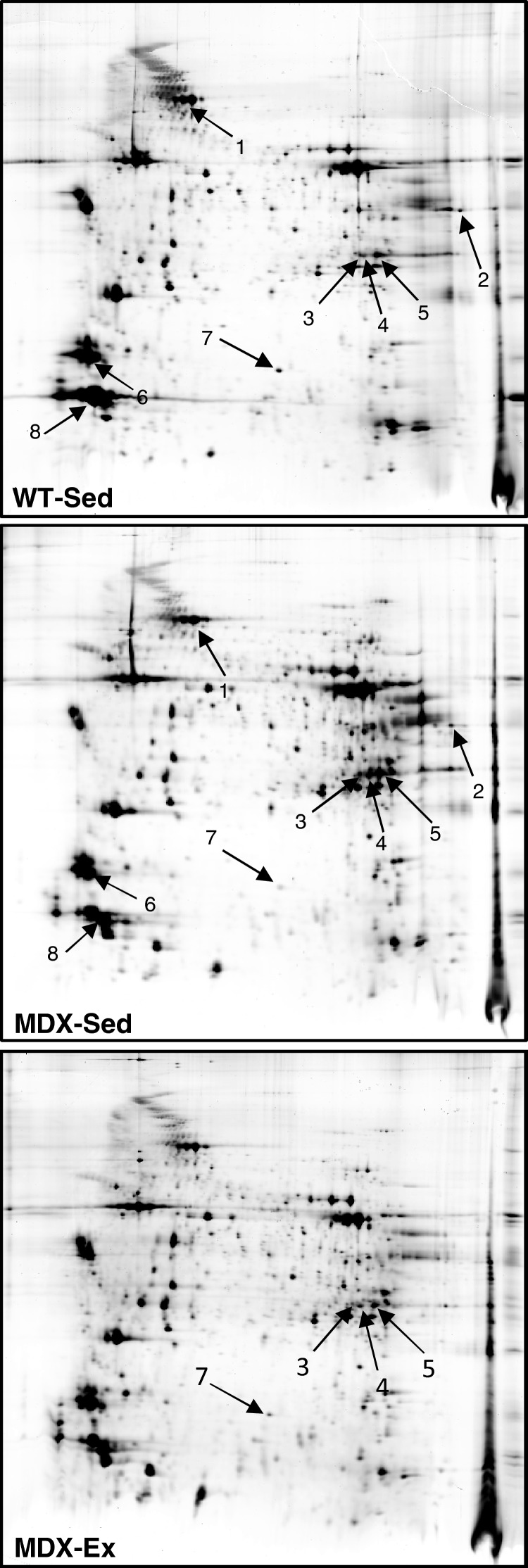
Detection of proteins differentially expressed in WT and MDX quadriceps Comparative analysis of protein profile of quadriceps from sedentary WT (WT-Sed) and sedentary *mdx* (MDX-Sed) mice highlighted eight protein spots showing a significant change in expression levels. These spots are marked by arrows and are numbered 1–8. The comparison of protein profile of quadriceps from MDX-Sed and exercised *mdx* (MDX-Ex) mice revealed that levels of spots 3, 4, 5 and 7 were affected by the low-intensity endurance exercise. Details of all these protein spots are reported in Table 1.

By comparing quadriceps from MDX-Sed and MDX-Ex mice, only four spots showed modulation in protein levels in response to the low-intensity endurance exercise. These spots were the three CA3 isoforms (pI 6.72/*M*_r_ 28122 Da; pI 6.74/*M*_r_ 27613 Da and pI 6.82/*M*_r_ 27782 Da) and the SODC (Figure 2).

The data concerning CA3 showed that protein levels of CA3 isoforms, found to be significantly up-regulated in quadriceps of MDX-Sed mice, were significantly or completely restored to WT-Sed values in quadriceps of MDX-Ex mice (Figure 3).

**Figure 3:**
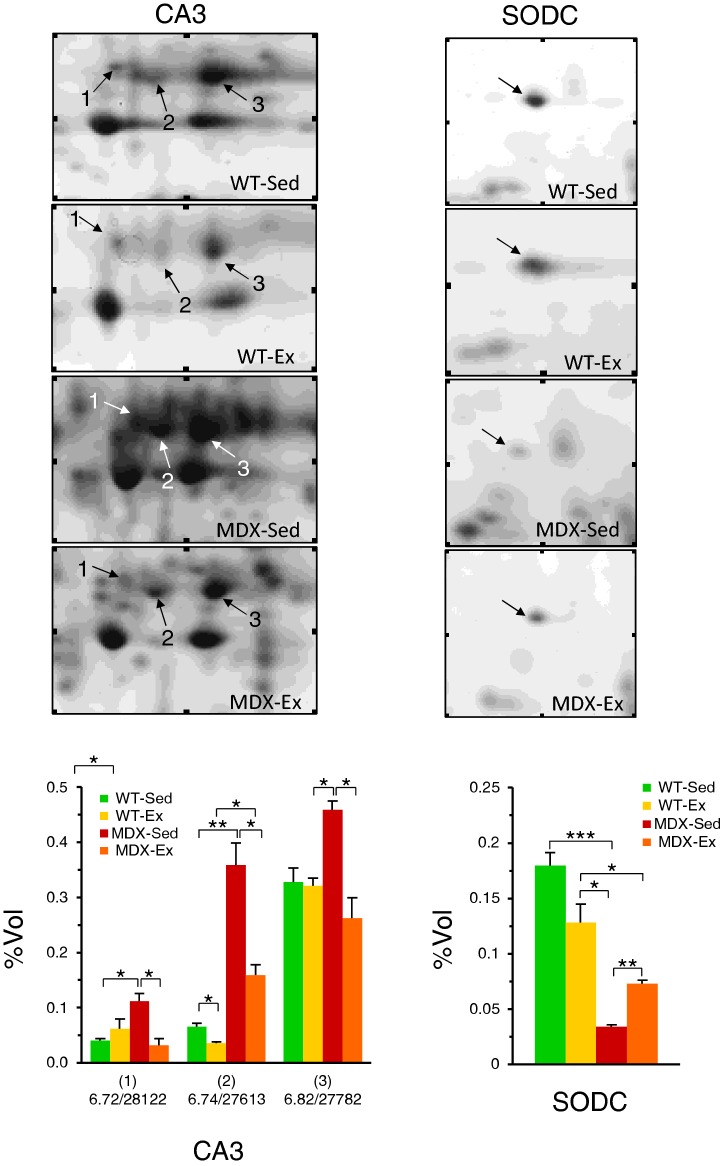
Expression pattern of CA3 isoforms and SODC in quadriceps Expanded view of 2D gels of quadriceps from sedentary WT (WT-Sed), exercised WT (WT-Ex), sedentary *mdx* (MDX-Sed) and exercised *mdx* (MDX-Ex) mice, in which the modulation of CA3 and SODC in MDX preparations can be observed. In the corresponding histogram, the %Vol values of each CA3 isoforms and SODC are reported. The CA3 isoforms are identified with pI-values and relative molecular masses: (1) pI 6.72/*M*_r_ 28122 Da; (2) pI 6.74/*M*_r_ 27613 Da and (3) pI 6.82/*M*_r_ 27782 Da. The values reported in the graphs are the mean of three independent experiments ± S.D. *t* test: *P<0.05; **P<0.01; ***P<0.001.

The data concerning SODC showed that the down-regulated protein levels found in quadriceps of MDX-Sed mice were significantly but not completely restored to WT-Sed values in quadriceps of MDX-Ex mice (Figure 3).

In order to evaluate if the low-intensity endurance exercise had specific effect on the quadriceps of dystrophic *mdx* mice, we assessed the expression levels of CA3 and SODC in WT-Ex mice. As reported in Figure 3, our analyses showed that no significant modulation of both CA3 and SODC was found in quadriceps of WT-Ex mice as compared with WT-Sed mice.

### Proteomic data validation by western blotting analysis

In order to validate key proteomic findings, comparative 1D and 2D WB analyses were carried out. As shown in Figure 4, western blot data showed that the levels of two CA3 isoforms, pI 6.72/*M*_r_ 28122Da and pI 6.74/*M*_r_ 27613 Da, are up-regulated in the quadriceps of MDX-Sed mice and the exercise restore them to WT-Sed basic levels, confirming data obtained with proteomic analysis. In contrast with proteomic analysis, CA3 isoform with pI 6.82/*M*_r_ 27782 Da was found unchanged in MDX-Ex mice as compared with MDX-Sed mice. Moreover, WB analysis of CA3 revealed the presence of a more acidic isoform of this enzyme, which was not detectable in the silver stained map. The values of pI and *M*_r_ (pI 6.62/*M*_r_ 28122 Da) of this CA3 isoform (Figure 4) were established by matching the WB with the corresponding silver stained maps. The levels of this isoform are significantly up-regulated in quadriceps of MDX-Sed mice in comparison with WT-Sed mice and appear restored to the levels of WT-Sed quadriceps in the MDX-Ex mice, with significant trend to be down-regulated (Figure 4). Similarly to proteomic analysis, WB analysis showed that there was no significant modulation of CA3 levels in WT-Ex mice as compared with WT-Sed (Figure 4).

**Figure 4:**
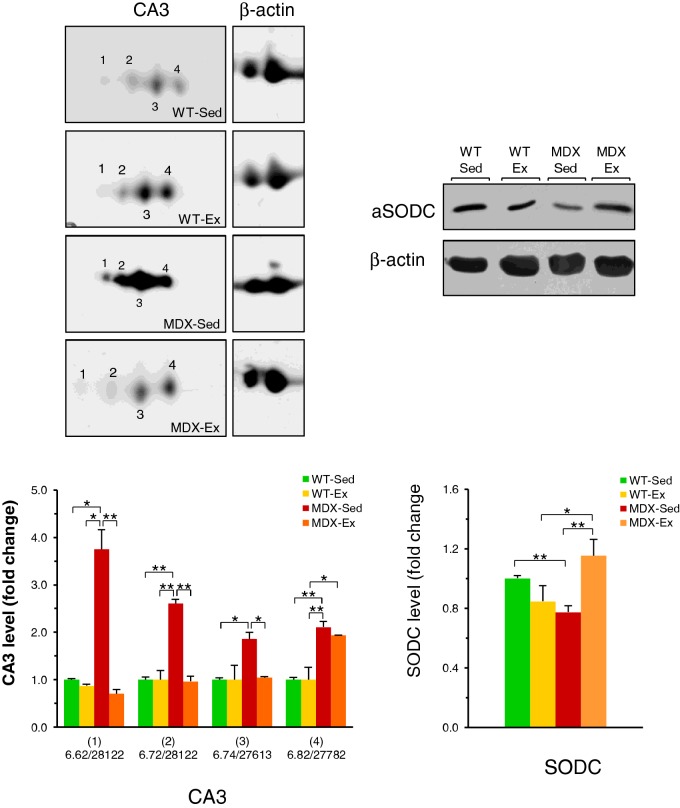
Western Blot of CA3 isoforms and SODC in quadriceps Representative immunoblots with expanded views of antibody-decorated protein spots and bands. Immunoblotting was performed after 2DE for CA3 and after 1DE for SODC. β-Actin was used as loading control. The blot densities are expressed as folds of control. CA3 isoforms are identified with pI-values and relative molecular masses: (1) pI 6.62/*M*_r_ 28122 Da; (2) pI 6.72/*M*_r_ 28122Da; (3) 6.74/*M*_r_ 27613 Da and (4) pI 6.82/*M*_r_ 27782 Da. Data are mean ± S.D.; *P<0.05; **P<0.01. Sedentary WT (WT-Sed), exercised WT (WT-Ex), sedentary *mdx* (MDX-Sed) and exercised *mdx* (MDX-Ex) mice.

The WB data of SODC showed that there is a significant reduction in SODC levels in the quadriceps of MDX-Sed mice compared with WT-Sed mice (Figure 4), confirming data obtained using proteomic analysis. These SODC levels found in MDX-Sed mice appeared completely restored to WT-Sed mice levels in MDX-Ex mice. However, whereas in WB the SODC levels found in MDX-Ex mice appeared completely restored to WT-Sed mice levels, they were only partially but significantly restored in proteomic analysis. WB analysis also confirmed proteomic data showing no significant modulation of SODC in WT-Ex mice as compared with WT-Sed mice (Figure 4).

## DISCUSSION

In the present study, with the proteomic procedure used, we have mapped few proteins as compared with data of previous studies [4,17,19,20] and therefore in the results obtained there is a restricted identification of proteins changed in MDX-Sed and MDX-Ex mice. However, despite the restricted number of proteins examined, we could identify some proteins with changed levels in MDX compared with WT mice and their modulation in MDX-Ex compared with MDX-Sed mice. In fact, among 17 proteins mapped we identified eight protein spots that were significantly changed in quadriceps muscle profiling of MDX-Sed mice, as compared with quadriceps of sedentary WT-Sed mice. The proteomic profiling suggests a dystrophy-associated alteration in various proteins of contractile apparatus [MLRS (myosin regulatory light chain 2 skeletal muscle isoform), MYH4 (myosin heavy chain 4), TnT (troponin T)], in proteins of oxidative stress (CA3, SODC) and ion homoeostasis [α/PVALB (parvalbumin α)]. All these altered proteins levels in *mdx* mice confirm previous available data [4,17–19].

### Comparative proteomic profiling: MDX-Ex mice compared with MDX-Sed mice

The aim of the present work was the identification of altered protein levels in MDX-Ex mice as compared with MDX-Sed mice in order to find explanation of strong reduction in degeneration muscle fibres in *mdx* mice induced by low-intensity endurance exercise [9]. Among the examined proteomic profiling, two types of proteins, CA3 and SODC, were significantly modified in MDX-Ex mice as compared with MDX-Sed mice. Indeed, levels of these proteins were already found dramatically changed in quadriceps of MDX-Sed mice, with CA3 up-regulated and SODC down-regulated, when compared with quadriceps of WT-Sed mice. Therefore, the comparative analysis revealed a statistically significant reduction in all four CA3 isoform levels and an increase in SODC levels in MDX-Ex mice as compared with respective protein levels found in MDX-Sed mice. All together these results highlight that exercise may restore to WT-Sed mice values the protein levels of CA3 and SODC found modified in muscle of MDX-Sed mice.

The increased level of CA3 isoforms in quadriceps of MDX-Sed mice and the strong decreased SODC protein levels suggest a state of oxidative stress in dystrophic muscle. On the other hand, the decreased levels of all isoforms of CA3 found in MDX-Ex mice and the parallel increased SODC protein level suggest improved muscle fibres response to oxidative stress. Since oxidative stress may contribute to pathophysiology of muscular dystrophy [21–23], the restored antioxidative response, especially the increased SODC levels, suggests a possible mechanism by which low-intensity endurance exercise may reduce muscle degeneration in *mdx* mice.

### Oxidative stress and SODC levels

A plethora of therapeutic strategies have been employed with the purpose of ameliorating dystrophin deficiency, including antioxidant therapeutics that may reduce damage and improve muscle function and quality of life [22,23]. The possibility that oxidative stress may contribute to muscle pathology is supported by experimental evidences that a deficit of antioxidant defence can lead to cellular dysfunction, damage and tissue degeneration [21]. Additional, an elevated oxidative stress has been also proposed as a contributing mechanism to muscle damage and weakness in dystrophin deficiency [21,22,24,25]. In view of this oxidative damage in dystrophic muscle, our findings showing that low-intensity endurance exercise may significantly recovery SODC levels in muscle of *mdx* mice suggest that a specific programme of training could contribute to dystrophic muscle recovery by promoting muscle protection against oxidative stress.

### Oxidative stress and CA levels

CAs are a family of zinc metalloenzymes which have various tissue distributions and intracellular locations in mammals [26,27]. These enzymes catalyse the interconversion between carbon dioxide and the bicarbonate ion and thus they are involved in crucial physiological processes connected with respiration and transport of CO_2_/bicarbonate between metabolizing tissues and lungs. CA3 is largely present in type I fibres of skeletal muscle and in less extent in type IIa and apparently absent from type IIb fibres [28,29].

As first evaluation of mean of the observed increase in all CA3 isoform protein levels in quadriceps of MDX-Sed mice, we considered the possible positive correlation with an increased need of acid-base balance regulation [30] and, based on this point of view, the increased levels of CA3 in *mdx* muscle should result protective against, e.g. pH change. However, in contrast with this possibility, it has been reported that treatment with CA inhibitors in muscular dystrophy reduces the degeneration/regeneration process [31]. This result suggests that high levels of CA3 could also be responsible for muscle degeneration, as also supported by data showing that CA inhibitors may be used as a therapeutic approach in some pathology in which CA is increased [26]. According to this prospective, the recovery of normal CA3 levels in MDX-Ex mice could mimic the therapeutic approach with CA inhibitors and therefore may contribute to the reduction in degeneration in dystrophic muscles.

As second evaluation of mean of the CA3 up-regulation in *mdx* muscle, we considered the possible protective role against oxidative stress. According to this, CA3 is largely present in slow twitch fibres with high oxidation potential and it has been suggested that CA3 may have a role in scavenging oxygen radicals and thereby protecting cells from oxidative damage [32,33]. Indeed, CA3 in skeletal muscle exists as a reservoir of oxidizable sulfhydryls that can be recruited to counteract acute and chronic oxidative insults [32]. Considering this potential role of CA3, the observed increase level of CA3 in MDX-Sed mice could correlate with elevated oxidative stress levels and therefore contribute to counteract muscle damage in *mdx* mice. Similarly, the reduction in CA3 levels in MDX-Ex mice could also be correlated to reduced oxidative stress by improving antioxidant mechanisms, such as the observed SODC protein level recovery and consequently less stimulus for CA3 as antioxidant protein. However, to better define a correlation between the reduced levels of CA3 in MDX-Ex mice and the recovery of damaged fibres-specific investigations are needed, since the reduction in CA3 levels in MDX-Ex mice could be secondary to muscle recovery itself.

### CA3 and SODC levels in WT-Ex mice

Comparative analysis of quadriceps from WT-Sed and WT-Ex mice did not show difference in both CA3 and SODC levels. The result that the low-intensity endurance training did not modify the CA3 levels is in agreement with other report [34] and could be correlated to the chosen low-intensity exercise protocol. In addition, although only speculative and therefore suggestion for future specific investigations, since CA3 is high expressed in slow twitch oxidative fibres [28,29], the unchanged CA3 levels in MDX-Ex mice indirectly may suggest a lack of significant shifting of fast compared with slow fibres sub-type following the low-intensity endurance training. On the other end, a lack of significant fibre-type shifting could be also supported by the data that in MDX-Ex mice CA3 levels were found reduced as compared with MDX-Sed mice.

Although previous data have reported that endurance exercise increases SODC levels [35], in the present study we did not find change in the SODC levels of WT-Ex mice. This result, as above discussed for CA3 levels, could depend on the low intensity endurance exercise used in our experiments and could correlate with absence or weak oxidative stress [36]. However, since in MDX-Ex mice, subject to the same training of WT mice, there was a significant recovery of SODC levels, we can't exclude a SODC gene stimulation in addition to recovery of damaged fibres that we observed in MDX-Ex mice [9].

## CONCLUSION

In conclusion, based on data found in the present study, it seems that low-intensity endurance exercise, by modulating some proteins involved in oxidative stress defence, may in part contribute to reduce, as observed in our previous study, cell degeneration in *mdx* muscles [9].

However, further investigations are needed to better define the extension of proteins change in MDX-Ex compared with MDX-Sed mice and its correlation with the recovery of damaged fibres in MDX-Ex mice.

## Abbreviations

2DE: 2D gel electrophoresis
CA3: carbonic anhydrase 3
DMD: Duchenne muscular dystrophy
*mdx*: mice with x-linked muscular dystrophy
MDX-Ex: exercised mice with X-linked muscular dystrophy
MDX-Sed: sedentary mice with X-linked muscular dystrophy
MLRS: myosin regulatory light chain 2 skeletal muscle isoform
MYH4: myosin heavy chain 4
α/PVALB: parvalbumin α
SODC: superoxide dismutase [Cu-Zn]
TnT: troponin T
WB: western blotting
WT: wild-type
WT-Sed: sedentary wild-type mice
WT-Ex: exercised wild-type

## References

[1] Matthews P.M., Benjamin D., Van B.I., Squier M.V., Nicholson L.V., Sewry C., Barnes P.R., Hopkin J., Brown R., Hilton-Jones D. (1995). Muscle X-inactivation patterns and dystrophin expression in Duchenne muscular dystrophy carriers. Neuromuscul. Disord..

[2] Hodgetts S., Radley H., Davies M., Grounds M.D. (2006). Reduced necrosis of dystrophic muscle by depletion of host neutrophils, or blocking TNFalpha function with etanercept in mdx mice. Neuromuscul. Disord..

[3] Ohlendieck K. (2011). Skeletal muscle proteomics: current approaches, technical challenges and emerging techniques. Skelet. Muscle.

[4] Lewis C., Carberry S., Ohlendieck K. (2009). Proteomic profiling of x-linked muscular dystrophy. J. Muscle Res. Cell Motil..

[5] Gardan-Salmon D., Dixon J.M., Lonergan S.M., Selsby J.T. (2011). Proteomic assessment of the acute phase of dystrophin deficiency in mdx mice. Eur. J. Appl. Physiol.

[6] Ge Y., Molloy M.P., Chamberlain J.S., Andrews P.C. (2003). Proteomic analysis of mdx skeletal muscle: Great reduction of adenylate kinase 1 expression and enzymatic activity. Proteomics.

[7] Odom G.L., Gregorevic P., Chamberlain J.S. (2007). Viral-mediated gene therapy for the muscular dystrophies: successes, limitations and recent advances. Biochim. Biophys. Acta.

[8] Guglieri M., Bushby K. (2010). Molecular treatments in Duchenne muscular dystrophy. Curr. Opin. Pharmacol..

[9] Frinchi M., Macaluso F., Licciardi A., Perciavalle V., Coco M., Belluardo N., Morici G., Mudo G. (2014). Recovery of damaged skeletal muscle in mdx mice through low-intensity endurance exercise. Int. J. Sports Med..

[10] Frinchi M., Di Liberto V., Olivieri M., Fuxe K., Belluardo N., Mudo G. (2010). FGF-2/FGFR1 neurotrophic system expression level and its basal activation do not account for the age-dependent decline of precursor cell proliferation in the subventricular zone of rat brain. Brain Res..

[11] Fontana S., Alessandro R., Barranca M., Giordano M., Corrado C., Zanella-Cleon I., Becchi M., Kohn E.C., De Leo G. (2007). Comparative proteome profiling and functional analysis of chronic myelogenous leukemia cell lines. J. Proteome. Res..

[12] Hochstrasser D.F., Patchornik A., Merril C.R. (1988). Development of polyacrylamide gels that improve the separation of proteins and their detection by silver staining. Anal. Biochem..

[13] Shevchenko A., Wilm M., Vorm O., Mann M. (1996). Mass spectrometric sequencing of proteins silver-stained polyacrylamide gels. Anal. Chem..

[14] Alessandro R., Gallo A., Barranca M., Principe S., Taverna S., Duro G., Cassata G., Becchi M., Fontana S., De Leo G. (2009). Production of an egg yolk antibody against Parietaria judaica 2 allergen. Poult. Sci..

[15] Yan J.X., Harry R.A., Wait R., Welson S.Y., Emery P.W., Preedy V.R., Dunn M.J. (2001). Separation and identification of rat skeletal muscle proteins using two-dimensional gel electrophoresis and mass spectrometry. Proteomics.

[16] Marouga R., David S., Hawkins E. (2005). The development of the DIGE system: 2D fluorescence difference gel analysis technology. Anal. Bioanal. Chem..

[17] Carberry S., Brinkmeier H., Zhang Y., Winkler C.K., Ohlendieck K. (2013). Comparative proteomic profiling of soleus, extensor digitorum longus, flexor digitorum brevis and interosseus muscles from the mdx mouse model of Duchenne muscular dystrophy. Int. J. Mol. Med..

[18] Ge Y., Molloy M.P., Chamberlain J.S., Andrews P.C. (2004). Differential expression of the skeletal muscle proteome in mdx mice at different ages. Electrophoresis.

[19] Matsumura C.Y., Menezes de O.B., Durbeej M., Marques M.J. (2013). Isobaric tagging-based quantification for proteomic analysis: a comparative study of spared and affected muscles from mice at the early phase of dystrophy. PLoS One.

[20] Doran P., Martin G., Dowling P., Jockusch H., Ohlendieck K. (2006). Proteome analysis of the dystrophin-deficient MDX diaphragm reveals a drastic increase in the heat shock protein cvHSP. Proteomics.

[21] Rando T.A. (2002). Oxidative stress and the pathogenesis of muscular dystrophies. Am. J. Phys. Med. Rehabil..

[22] Kim J.H., Kwak H.B., Thompson L.V., Lawler J.M. (2013). Contribution of oxidative stress to pathology in diaphragm and limb muscles with Duchenne muscular dystrophy. J. Muscle Res. Cell Motil..

[23] Whitehead N.P., Pham C., Gervasio O.L., Allen D.G. (2008). N-acetylcysteine ameliorates skeletal muscle pathophysiology in mdx mice. J. Physiol..

[24] Whitehead N.P., Yeung E.W., Froehner S.C., Allen D.G. (2010). Skeletal muscle NADPH oxidase is increased and triggers stretch-induced damage in the mdx mouse. PLoS One.

[25] Mendell J.R., Engel W.K., Derrer E.C. (1971). Duchenne muscular dystrophy: functional ischemia reproduces its characteristic lesions. Science.

[26] Supuran C.T., Scozzafava A. (2007). Carbonic anhydrases as targets for medicinal chemistry. Bioorg. Med. Chem..

[27] Pastorekova S., Parkkila S., Pastorek J., Supuran C.T. (2004). Carbonic anhydrases: current state of the art, therapeutic applications and future prospects. J. Enzyme Inhib. Med. Chem..

[28] Nishita T., Yorifuji D., Orito K., Ichihara N., Arishima K. (2012). Muscle carbonic anhydrase III levels in normal and muscular dystrophia afflicted chickens. Acta Vet. Scand..

[29] Fremont P., Charest P.M., Cote C., Rogers P.A. (1988). Carbonic anhydrase III in skeletal muscle fibers: an immunocytochemical and biochemical study. J. Histochem. Cytochem..

[30] Supuran C.T., Scozzafava A., Casini A. (2003). Carbonic anhydrase inhibitors. Med. Res. Rev..

[31] Giacomotto J., Pertl C., Borrel C., Walter M.C., Bulst S., Johnsen B., Baillie D.L., Lochmuller H., Thirion C., Segalat L. (2009). Evaluation of the therapeutic potential of carbonic anhydrase inhibitors in two animal models of dystrophin deficient muscular dystrophy. Hum. Mol. Genet..

[32] Zimmerman U.J., Wang P., Zhang X., Bogdanovich S., Forster R. (2004). Anti-oxidative response of carbonic anhydrase III in skeletal muscle. IUBMB Life.

[33] Raisanen S.R., Lehenkari P., Tasanen M., Rahkila P., Harkonen P.L., Vaananen H.K. (1999). Carbonic anhydrase III protects cells from hydrogen peroxide-induced apoptosis. FASEB J..

[34] Zoll J., Ponsot E., Dufour S., Doutreleau S., Ventura-Clapier R., Vogt M., Hoppeler H., Richard R., Fluck M. (2006). Exercise training in normobaric hypoxia in endurance runners. III. Muscular adjustments of selected gene transcripts. J. Appl. Physiol..

[35] Hollander J., Fiebig R., Gore M., Bejma J., Ookawara T., Ohno H., Ji L.L. (1999). Superoxide dismutase gene expression in skeletal muscle: fiber-specific adaptation to endurance training. Am. J. Physiol..

[36] Laughlin M.H., Simpson T., Sexton W.L., Brown O.R., Smith J.K., Korthuis R.J. (1990). Skeletal muscle oxidative capacity, antioxidant enzymes, and exercise training. J. Appl. Physiol..

